# Local Error Estimates Dramatically Improve the Utility of Homology Models for Solving Crystal Structures by Molecular Replacement

**DOI:** 10.1016/j.str.2014.11.020

**Published:** 2015-02-03

**Authors:** Gábor Bunkóczi, Björn Wallner, Randy J. Read

**Affiliations:** 1 Department of Haematology, Cambridge Institute for Medical Research, University of Cambridge, Hills Road, Cambridge CB2 0XY, UK; 2 IFM, Linköping University, S-581 83 Linköping, Sweden

## Abstract

Predicted structures submitted for CASP10 have been evaluated as molecular replacement models against the corresponding sets of structure factor amplitudes. It has been found that the log-likelihood gain score computed for each prediction correlates well with common structure quality indicators but is more sensitive when the accuracy of the models is high. In addition, it was observed that using coordinate error estimates submitted by predictors to weight the model can improve its utility in molecular replacement dramatically, and several groups have been identified who reliably provide accurate error estimates that could be used to extend the application of molecular replacement for low-homology cases.

## Highlights

• Error estimates increase the value of homology models for molecular replacement
• Poorer models with good error estimates trump better models without errors
• A simple protocol creates coordinate error estimates for individual models
• Local coordinate error estimates enable molecular replacement for more targets

## Teaser

Although estimating the size of local errors in homology models is not common practice, Bunkóczi et al. show that the provision of error estimates can have a substantial practical impact in the utility of these models when used to solve crystal structures by molecular replacement.

## Introduction

About two-thirds of crystal structures deposited in the Protein Data Bank (PDB) (Berman et al., 2003) are now solved by the method of molecular replacement (MR), and it has been estimated that about 80% could have been solved by MR using templates available at the time of deposition (Long et al., 2008). Traditionally, MR has been the method of choice when there is a template with a sequence identity greater than 30%–40%. If such a sequence identity threshold could be pushed down, MR could be applied even more widely. For this reason, there has been significant interest in the application of homology modeling to improve templates prior to MR. As recently as 5–10 years ago, the perception was that homology modeling tended to make templates worse instead of improving them, but in the last few years it has become apparent that homology modeling can now add value to the templates, making them better models for MR. This has become an active area of research and several pipelines have been developed for ready use, e.g., mr_rosetta (DiMaio et al., 2011) in the PHENIX package (Adams et al., 2010).

In CASP7, Read and Chavali (2007) introduced an MR score to judge the quality of models submitted to the high-accuracy template-based modeling category. The scores explored were the log-likelihood gain (LLG) computed for the best potential MR solution found by the MR program Phaser (McCoy et al., 2007) and the Z score of the top correct solution (if any). One sobering result was that, of 1,588 models evaluated, only 33 (2.1%) proved to be better for MR than the best available template. In retrospect, however, the high-accuracy template-based modeling category was least likely to reveal an improvement from homology modeling, as one of the criteria for entry was that there was already a good template!

At the time, computing these scores was computationally prohibitive because a complete MR search had to be carried out for each model, so this scoring procedure was not applied at the time to models from other categories of CASP. Unfortunately, for this purpose, the MR search as implemented in Phaser is adaptive; the poorer the model, the longer the search takes, with models that are too poor to find a solution taking the longest. More recently, a fast procedure has been developed to calculate the LLG scores for any models, given the availability of a reasonably good solution onto which the models can be superposed (typically, the target structure). Augmented with the increase in available computing power, this enabled a large-scale evaluation of predictions from the CASP10 experiment, including models from all categories.

Read and Chavali (2007) suggested that success in MR might be improved by translating estimates of coordinate uncertainty into an inflation of the crystallographic B factors, to smear the atoms in the model over their range of possible positions. This suggestion was taken up by Pawlowski and Bujnicki (2012), who showed that perfect knowledge of coordinate errors would have a very large impact on MR success and that the use of estimated coordinate errors could have a smaller but still significant impact. Here we show that the best model quality assessment algorithms indeed add substantial value to MR models, even when only a single model is available.

## Results and Discussion

### Quick LLG Calculation

There are several problems with performing a full-scale MR search to calculate the LLG score for an arbitrary model. First, it can be very time consuming, especially if the unit cell contains several copies of the molecule. Second, if the correct solution is not found, the resulting LLG value is not valid. In addition, an automatic test to check whether a solution has been found or not depends on arbitrary cutoffs and decisions, e.g., whether to keep or discard partially correct solutions.

Targets for the CASP experiments all have known structures and, although these are kept secret from predictors they are used for assessment. Therefore, the predicted structure can be superposed onto all copies of the target chain and an LLG score can be calculated. Since structural superposition does not in general yield identical results to those based on electron density, an additional positional refinement is necessary to obtain the best fit. It is important to note that if no meaningful superposition can be made, the initial structure will not be within convergence radius of refinement and the resulting score will be invalid. In addition, for structurally periodic targets and an imprecise superposition, refinement can move the structure out of register, also resulting in an invalid score.

The LLG calculation is also dependent on the assumed error (variance root mean square [vrms]), which is normally estimated based on the sequence identity. For predicted structures, an estimate could be calculated from superposing them onto the true structure; however, this value will still need to be refined to obtain a best fit, and the vrms refinement in Phaser (McCoy et al., 2007) does not need a precise estimate for convergence. The refinement can sometimes terminate prematurely, since the LLG landscape can contain multiple maxima, but this only seems to happen in about 0.5%–1% of cases, and these can easily be identified from GDT_TS versus LLG score plots. Restarting refinement from another starting value usually results in a valid score.

### Correspondence with Other Metrics

**Figure 1:**
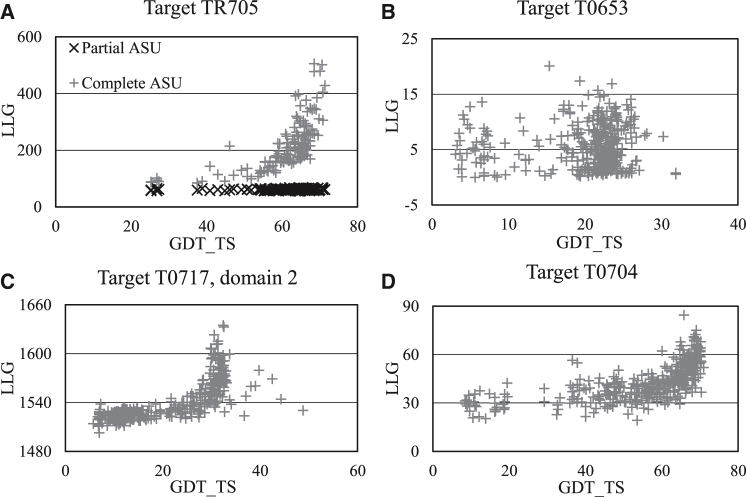
Typical LLG versus GDT_TS Scatter Plots Observed for Targets (A) Target TR705 contains two domains and refinement of one of these was requested. If the second domain is not taken into account in the likelihood calculations, the black curve is obtained, which shows no correlation between the two scores. However, by taking the contribution from the second domain into account (grey curve), a clear correlation is obtained (for scores shown, the contribution of the second domain alone is subtracted for the plot). ASU, asymmetric unit. (B) Uninformative LLG plot for target T0653 with all models falling into the low accuracy zone. (C) Very sensitive LLG plot for target T0717, domain 2 (taking the unpredicted domain 1 into account). Predictors have managed to model residues Val67 to Gly119 (out of 166 residues) very accurately, and this gives a clear signal in scoring with the 1.9 Å X-ray data. For the “outlier” models above GDT_TS = 35, the accuracy of the named residue segment is comparable with that of the rest of the structure. (D) Atypically small signal observed for target T0704.

LLG scores were calculated for all predictions submitted for CASP10 targets that were determined by X-ray crystallography and for which the measured X-ray diffraction amplitudes were available. The relationship with the GDT_TS score was examined on scatter plots (Figure 1). For the majority of cases, the LLG score showed a clear functional relationship with the GDT_TS score. At GDT_TS < 40–50, the LLG scores were almost constant (average Spearman correlation coefficient for segment GDT_TS ≤ 50, 36%); they started to increase slowly for GDT_TS values above 50–60 and then very rapidly for GDT_TS > 80 (average Spearman correlation coefficient for segment GDT_TS > 50, 71%), although some deviations have also been observed (Figures 1C and 1D). This suggests that the LLG score cannot discriminate among predictions with large errors but can accurately rank good-quality predictions.

This functional relationship between the two scores is not unexpected. Both the GDT_TS and the LLG measure deviations from a reference structure and, unlike root-mean-square deviation (rmsd), the penalty given to deviations is limited. This limit is imposed in GDT_TS by a series of cutoff values and in LLG by a smooth function deriving from the difference between the observed and calculated structure factor values in the presence of errors (Read, 1990), which does not continue to degrade once errors are large compared with the resolution of the diffraction data. However, there are also important differences between the two. The LLG score depends on the measured X-ray data and therefore is also affected by the resolution of the structure. It is an all-atom score and therefore downweights pure Cα predictions on the basis of low completeness. Nonetheless, even relatively small fragments can receive significant LLG scores if the prediction is very accurate.

Although the LLG score measures the composite effect of the accuracy and completeness of predicted structures, it is also possible to describe their accuracy alone using MR calculations. This is expressed in the refined vrms value, which is independent of the completeness. The vrms is an effective rmsd value that calibrates the likelihood functions, based on the level of agreement between observed and calculated structure factors that would be obtained if the errors in all the atomic positions were drawn from the same gaussian error distribution. If the errors were drawn from a gaussian distribution, the vrms would be equivalent to the rmsd but, compared with the rmsd, the effects of outliers are downweighted. Therefore, for predictions with approximately the same completeness, a clear negative correlation can be found between GDT_TS and vrms, which is approximately linear. In addition, for predictions that are reasonably complete, vrms shows a linear relationship with the common rmsd from Local-Global Alignment (LGA) (Zemla, 2003).

LLG scores of predicted structures are only comparable if calculated against the same X-ray data set. The LLG score can therefore not be used to evaluate cross-target performance of predictors, so two indicators based on the LLG score have been selected for this purpose. The first of these is the common Z score calculated for each target, which measures how well a predictor is performing with respect to others. Because of the standard deviation in the denominator of the Z score, it gives more weight to targets for which most predictors submitted similar quality models. The individual Z scores of predictions are then averaged for each group. The second score measures the improvement with respect to a suitably chosen baseline model, and gives more weight to targets where the baseline has low quality. This is referred to as improvement score (I score, defined in Equation 4 below), and is calculated using the best prediction of the group for a given target only.

### Accounting for Model Errors

The accuracy of a structure as an MR model often varies along the chain. In general, it is the highest in the core and lowest on the protein surface. Methods to estimate model quality are evaluated as part of the CASP exercises, and several such methods have been shown to give reasonably reliable estimates of local coordinate accuracy (Kryshtafovych et al., 2014). It is possible to take this predicted variation in accuracy into account for the MR calculation by manipulating the atomic displacement parameters (B factors) of constituent atoms (Read, 1990), incrementing the B factors by an amount proportional to the expected positional error squared as defined in Equation 1, where |Δr| is the absolute error in angstroms:

(Equation 1) $$\Delta B=\frac{8\pi^{2}}{3}\langle {\left|\Delta r\right|}^{2}\rangle$$

Note that Pawlowski and Bujnicki (2012) omitted the factor of 3 in the denominator, which may have reduced the size of improvements they observed. This factor (frequently omitted or poorly explained in the crystallographic literature) is required to account for the fact that the component of the mean-square coordinate error in any particular direction (specifically, in this case, parallel to the diffraction vector) is one-third of the overall mean-square coordinate error for an isotropic distribution of error.

The error estimates provided with the models by CASP predictors were used to establish whether MR results could be improved by taking them into account, and at the same time whether the error estimates are accurate enough for this to have a measurable effect. The average improvement found is rather modest; however, this can be attributed to the fact that the majority of predictors do not actually submit error estimates. When the average is calculated for predictors TS026 (ProQ2clust), TS130 (Pcomb), TS273 (IntFOLD2), TS280 (ProQ2clust2), TS285 (McGuffin), TS388 (ProQ2), and TS498 (IntFOLD), which were judged (as discussed below) to have submitted meaningful error estimates, the improvement in the LLG score is a staggering 25% with respect to the same models with constant B factors applied throughout the chain. This considerable improvement in model quality suggests that success of MR could be vastly enhanced if error estimates were taken into account, in agreement with the results from Pawlowski and Bujnicki (2012). To judge the effect of omitting the factor of 3 from Equation 1, LLG scores have been recalculated for the aforementioned groups using the formula of Pawlowski and Bujnicki (2012). This has resulted in an average LLG score almost 10% lower than with Equation 1, although with a large variability, and sometimes the “wrong” formula gave better results. However, it is important to note that Phaser requires the errors to be on an absolute scale, and scale-factor errors in prediction methods could account for occasional deviations from the theory. Multiple calculations involving different scale factors would quite possibly improve results even further but this was not explored.

All the predictors submitting meaningful error estimates were using a specified model quality assessment program (MQAP) to predict the model error. The MQAPs ModFOLD3 (McGuffin and Roche, 2010) and ModFOLD4 (McGuffin et al., 2013) were used to predict errors in models from IntFOLD (Roche et al., 2011) and IntFOLD2 (Buenavista et al., 2012), respectively. ProQ2, ProQ2clust, and ProQ2clust2 (Ray et al., 2012) as well as Pcomb (Wallner and Elofsson, 2006) are all MQAPs that were used to predict errors in models submitted to the server category of CASP.

### Refinement Targets

For a refinement target, a starting model is provided by the organizers and predictors are asked to improve it. However, since the best refinement models did not contain useful error estimates, these were not considered for this category (data not shown). On the other hand, the given starting model establishes a well-defined base level that can be used to measure the improvement in the structure.

There were 13 refinement targets assigned with X-ray data available. In all cases, the best prediction was of higher quality than the starting model, sometimes considerably. On average, the best prediction had a 30% higher LLG score than the starting model. On the other hand, only about 20% of all predictions were better than the starting model.

Average prediction quality has been calculated for predictors that submitted models for at least seven targets. Based on this measure, the best-performing predictors are TS049 (FEIG; Mirjalili et al., 2014), followed by TS197 (Mufold; Zhang et al., 2010), which improve the starting model in terms of the LLG score by 40% or 30%, respectively, followed by numerous others around the 10% mark.

Since there was very little variation in the extent of modeled regions, with almost all predictors predicting the full structure requested, the LLG scores showed a very clear correlation with the GDT_TS score. In addition, the vrms showed a linear relationship with the GDT_TS score (with a negative slope). Predictions that did not obey this latter relationship were of lower completeness; e.g., side chains or whole loops were missing.

### Template-Based Modeling Targets

Out of 97 template-based modeling (TBM) targets, 68 had X-ray data available. All models submitted by predictors were evaluated if they could be meaningfully superposed onto the target. Predictions were evaluated with three B-factor schemes: (a) B factors as present in the PDB file, (b) B factors calculated assuming that the submitted values are expected errors, using Equation 1, and (c) constant B factors.

As can be expected, results were more diverse than for the refinement targets. For several targets, most predictions were below the quality requirements of the LLG score and were given a flat nondiscriminative LLG score.

Since the templates used by predictors for a particular TBM target are not necessarily known, this presented a challenge to establish a baseline for model quality. Therefore, archived results from HHPRED (Söding et al., 2005) searches conducted on the day the target was released for predictions were used to determine which templates would have been available. Homologs found by the search were processed using the default protocol of Sculptor (Bunkóczi and Read, 2011) using the HHPRED alignment. This corresponds to a typical workflow in macromolecular crystallography, and the quality of the models is close to what would routinely be used. LLG scores were calculated for all of these, and the best template was selected and used as a basis for comparison. On the one hand this procedure cannot use information from multiple good-quality homologs and could be outperformed by modeling protocols but, on the other hand, predictors were not able to evaluate their templates with the experimentally observed structure factor amplitudes.

Of the 68 evaluated targets, a prediction better than the best available template was submitted for 30. On average, the best prediction was 30% better in quality than the best template (including targets where the best predictions were worse than the best template), indicating that for the best prediction the improvement is more often than not higher than the average loss of quality. On the other hand there were many poor predictions and only 1,680 of the evaluated 26,421 predictions were better than baseline (6.4%). However, significant variability was observed among predictors and this is illustrated for a selected set of groups in Table 1.

#### Identifying Error Estimates

**Figure 2:**
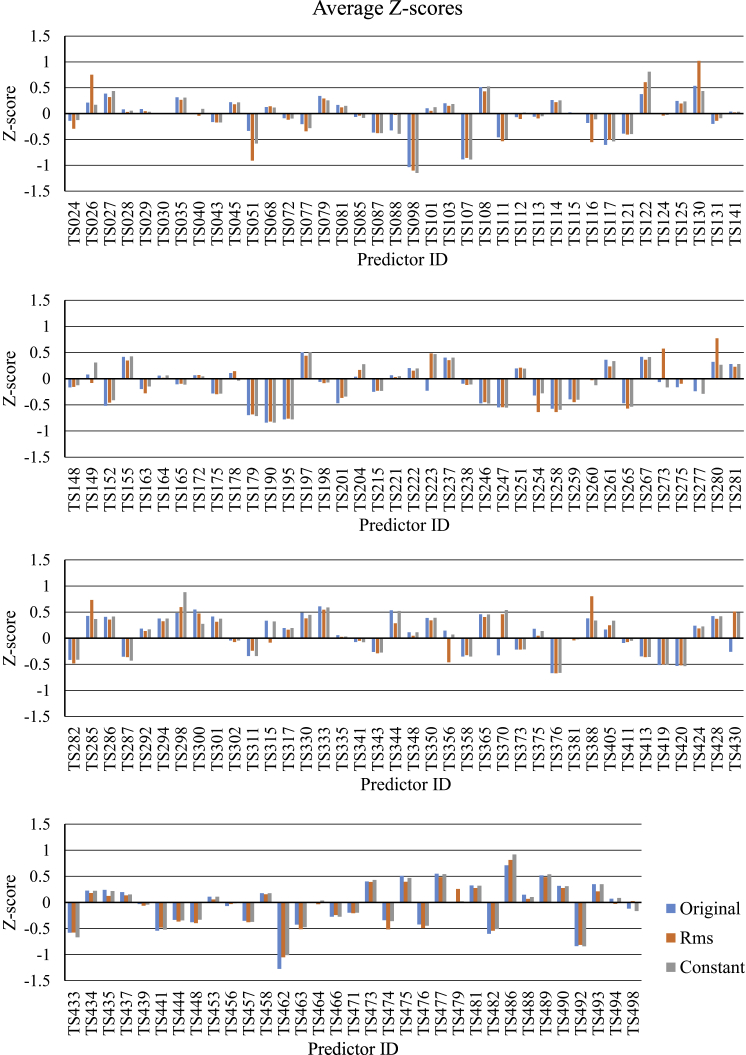
Average Z Scores for Predictors Calculated with All Three B-Factor Schemes In the original scheme, the numbers appearing in the B-factor field were used as is; in the root mean square (Rms) scheme, these were converted into a B factor using Equation 1 and, in the constant scheme, these were set to a constant number.

Predictors are asked to submit error estimates in the B-factor column along with the predicted coordinates. However, the submitted values are often zeros or actual B factors carried over from the template, and there is no explicit indication of how the B-factor column should be interpreted. To identify predictors submitting meaningful error estimates, the average Z scores were calculated for all three B-factor evaluation schemes (Figure 2). Assuming a predictor either submits error estimates with all predictions or none of them, it was expected that the average Z score for the B-factor scheme assuming error estimates (scheme (b) above) should be higher than for the other two, and tentatively a cutoff Z score difference of 0.1 was used. This highlighted 11 predictors. However, for one of these (TS311, Laufer), only three data points were available so this group was removed from the list. For the others, an additional check was performed by calculating the frequency with which the highest scoring prediction for a particular target was calculated with B-factor scheme (b). Results in Table 1 show that although the majority of the highest scores are achieved when interpreting the submitted numbers as error estimates, this is not exclusively the case. A potential explanation for this could be that the LLG score does not discriminate among low-quality predictions, hence the resulting ranking is not reliable.

It is instructive to consider which B-factor scheme yielded the best prediction for each target. In 37 of 68 cases, the best model was calculated with values interpreted as B factors, as in scheme (a) above; in 24 cases, the best model was calculated with the values interpreted as rmsd, as in scheme (b); while in the remaining seven cases the best model used a constant B factor. Considering that of the 147 participants potentially only 11 predictors submitted error estimates, this also suggests that making use of these estimates dramatically improves the quality of the resulting models for MR.

#### Molecular Replacement Performance

First, to evaluate the structural accuracy of predictions, the I scores calculated with constant B factors were used. The best-performing group is TS028 (YASARA), with an overall I score of −0.183, followed by TS330 (BAKER_ROSETTASERVER) with an I score of −0.186. Although these numbers indicate that on average the template has been degraded, it is important to note that the baseline model was selected based on its LLG score, which in general could not be calculated without access to diffraction data.

Second, to evaluate the composite effect of structural accuracy and atomic error predictions, the same procedure was performed, but now taking the best LLG score from B-factor scheme (b). Groups not submitting any error predictions received the same score as before. However, substantial improvements were observed for the ten groups mentioned above. The best-performing group is now TS130 (Pcomb), with an overall I score of −0.098, and the second-best group is TS280 (ProQ2clust2) with −0.122. The average improvement from incorporating atomic error estimates, for the groups submitting them, is 0.11 I score units. Conveniently, the overall scores for the best structure-only predictors, TS028 (YASARA) and TS330 (BAKER_ROSETTASERVER), do not change significantly on changing the B-factor scheme. However, four groups of the ten that provide error estimates now have a better overall score than the best structure-only predictor.

**Figure 3:**
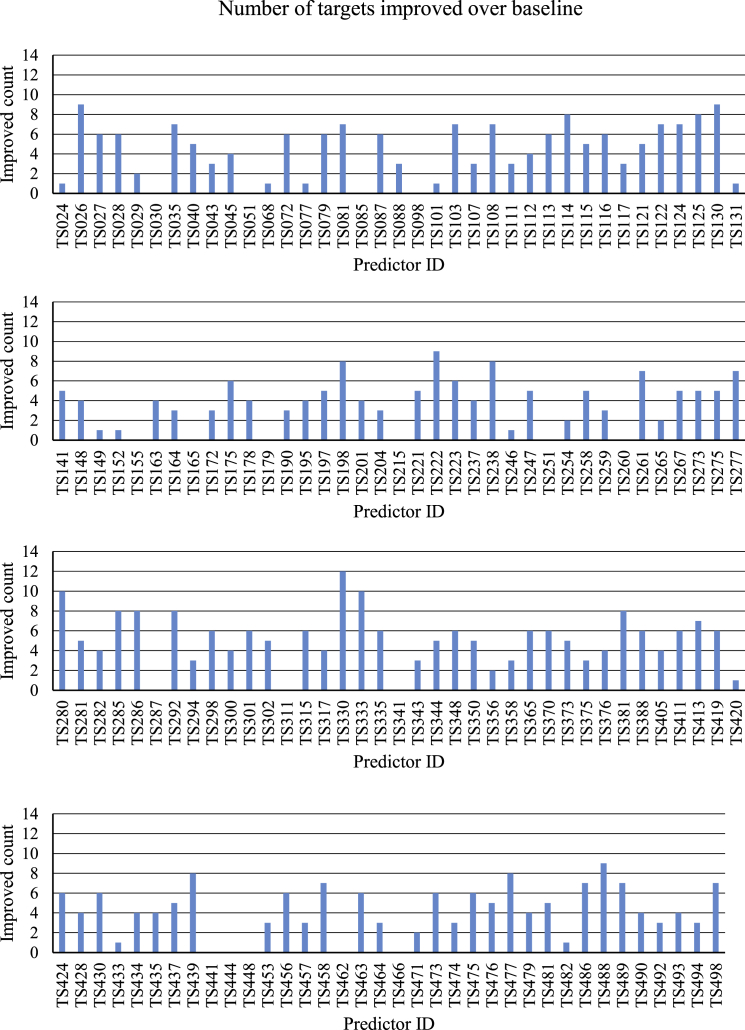
Number of Targets Improved upon the Baseline Structure, Taking into Account All Three B-Factor Schemes

Third, it was evaluated how many times each group managed to improve upon a particular baseline template. For this calculation, all B-factor schemes were taken into account and, for each target, the highest overall scoring model was selected. The best-performing group with 12 improvements was TS330 (BAKER_ROSETTASERVER), followed by TS280 (ProQ2clust2) and TS333 (MUFOLD-Server; Zhang et al., 2010), with ten improvements each (Figure 3).

### Weighting Molecular Replacement Models by Error Estimates

A set of 20 non-CASP borderline MR test cases were selected, in which the correct solution appears in the list of possible solutions, but not as the best hit. For these cases, alignments were generated using the structural alignment program LSQMAN (Kleywegt et al., 2001) with a fairly generous cutoff (8 Å) for generating the alignment from the structural superposition, so that the resulting alignment could be considered as the best possible using an ideal sequence-alignment tool without any structural information. However, for comparison, sequence alignments were also calculated with MUSCLE (Edgar, 2004). Using these alignments, homology models were created using SWISS-MODEL (Biasini et al., 2014), based on the template structures originally used as MR models. This step was required for accurate error prediction, since the actual sequence has to be mapped onto the structure and side chains have to be present. SWISS-MODEL was selected for this calculation because of ease of use. The local errors of these models were predicted by ProQ2 (Ray et al., 2012), converted into B factors, then mapped onto the corresponding MR model, which was generated from the same template and the same alignment using Sculptor (Bunkóczi and Read, 2011). The LLG score was calculated for the resulting model using constant B factors and ProQ2-error-based B factors. Improvement scores for these B-factor weighted models are shown in Table 2.

Interestingly, the average improvement with both the structural and sequence-based alignments was similar (32% and 28%, respectively). However, improvements with structural alignments seem to be more consistent, with 17 of 20 MR models having improved (15 of 20 with the sequence-based alignment), and the worst “improvement” being −10% (−27% with the sequence-based alignment).

Improvements were also calculated against MR models used with original B factors from the template structure, since this MR model is closer to that routinely used by crystallographers. In this case, structural alignments were found clearly to be superior to sequence-based alignments, with the average improvement being 34% versus 19%; 17 of 20 models had still improved with the structure-based alignment, but this declined to 14 of 20 for the sequence-based alignment. On the other hand, this indicates that even with relatively crude alignments, an average 20% improvement can be expected in MR if predicted errors are taken into account. In addition, although it is not possible to reach the accuracy of structural alignments when no structure is available, modern profile-profile methods can come fairly close, and a 25% improvement on average is potentially realistic.

### Conclusions

#### The LLG Score

The LLG score provides a direct measure for evaluating the quality of a predicted structure for MR. However, based on the experience presented, it can rank only relatively high-quality predictions, with a GDT_TS above approximately 60.

The correspondence between the GDT_TS and the difficulty of MR has been noted previously (Giorgetti et al., 2005). As the difficulty of MR is proportional to the discriminative power of the LLG score for correct solutions versus noise, it is clear that MR is unlikely to work with models having a GDT_TS below 40–50, since the LLG score is broadly flat in that region, at least for molecules of the size typically explored in CASP.

The current procedures allow this metric to be applied only for structures determined by X-ray crystallography. To the extent that we are interested in evaluating the utility of template-based models for practical use in MR, this is not a limitation. However, it restricts the applicability of this score to all targets in the CASP context. In principle, it would be possible to calculate X-ray data for structures determined by other methods, and simulate an X-ray structure that could participate in LLG scoring. The synthetic data would probably yield higher LLG scores, as there are effects in real data that are difficult either to account for in the structural model or to simulate in synthetic data, such as the effects of anharmonic motion or lattice imperfections. Nonetheless, Z scores would allow the quality of different models to be compared with scores for real data on a similar scale.

With synthetic data, one limitation of the LLG score as a criterion for CASP could be removed. The LLG score depends not only on the accuracy of the atomic coordinates, which are modeled by predictors, but also on the accuracy of the B-factor distribution along the chain, which is not modeled. Synthetic data could be computed with constant B factors, removing their influence from the LLG scores. In this way, the submitted error estimates should purely account for structural deviations between target and prediction, while in the current setup these may partially compensate for the missing B factors. However, in our experience, the effect of B-factor differences between model and target in the LLG score is only measurable for highly accurate models. For distant models, B factors seem to play a larger role in weighting parts of the model according to their respective errors.

#### Model Error Estimates

It has been found that when error estimates are available and accurate, they allow the calculation of appropriate model weights that result in a higher LLG score. As this score is a good descriptor for the difficulty of the MR search that would be conducted if an unknown structure were to be solved with the model, a higher LLG score will translate into a higher success rate in MR.

It is interesting that no structural improvement is necessary for the model to achieve a higher score. In fact, predictors would not be expected to achieve a perfect agreement with the experimental coordinates, since the structure can be influenced by crystal packing. However, by identifying segments that are highly flexible and are the most likely to adopt a different conformation in the crystal, these could be weighted down accordingly, which would improve a model's applicability for MR.

The utility of error estimates in MR has been investigated by Pawlowski and Bujnicki (2012), who reported improvements with errors derived from consensus methods. This finding is in contrast to our results, whereby single-model methods such as ProQ2 (Ray et al., 2012) perform comparably with consensus methods. The difference in their results may have arisen partly from the omission of the factor of 3 in Equation 1, which has been shown to yield lower LLG values, or from differences in the software used for MR calculations.

It seems to be a recurring finding in CASP assessment that predictors fail to assign realistic confidence estimates to their predictions, except for a very few groups (Mariani et al., 2011). Although it is difficult to predict coordinate errors reliably, the current situation could also be a consequence of assessing different metrics of the prediction in isolation. The LLG score offers a metric that is able to measure the cumulative quality of both the structure and the error estimates. More importantly, it offers a concrete measure of how accurate error estimates make a model more useful.

#### Prediction of Coordinate Errors

In principle, there are two strategies to obtain coordinate error estimates in a model; one using consensus (Wallner and Elofsson, 2005) and one using information only from the model itself, i.e., so-called single-model methods (Wallner and Elofsson, 2006). The consensus methods use as input an ensemble of models, usually constructed using different techniques. The error estimate for a given model is obtained by calculating the average coordinate error after superimposing the model on all models in the ensemble. It is also possible to obtain coordinate error predictions for other models by including them in the ensemble; methods applying this approach are sometimes referred to as quasi-single. However, since these models still rely on an ensemble, they are effectively a consensus method. Pure single-model methods, on the other hand, use only information from the model itself to calculate the error, and in this respect they are more similar to a regular energy function. The best single-model methods, such as ProQ2 used in this study, integrate different features, such as agreement between predicted and actual secondary structure and predicted and actual residue surface area, with regular knowledge-based potentials based on amino acid and atom type contact preference calculated from known structures or models (Ray et al., 2012). In general, the consensus methods have a higher accuracy, but, as shown in this study, the single-model method ProQ2 produces results similar to those of the best consensus methods (e.g., Pcomb and IntFold). In addition, at least for Pcomb, the model quality assessment was performed on exactly the same set of models.

#### Improving Molecular Replacement Procedures

During large-scale evaluation of predictions submitted to CASP10, it has become apparent that a previously neglected source of information, namely coordinate error estimates, can be used to improve MR protocols. In addition, currently existing algorithms used in modeling for the prediction of coordinate errors have been found to be sufficiently accurate to be highly useful. By introducing such error estimates into MR pipelines, such as MrBUMP (Keegan and Winn, 2007) and BALBES (Long et al., 2008) in the CCP4 package (Winn et al., 2011) and MRage (Bunkóczi et al., 2013) in the PHENIX package (Adams et al., 2010), the success rate of MR should be further improved.

In addition to improving the utility of theoretical models in MR by incorporating error estimates, first reported by Pawlowski and Bujnicki (2012), it has been found that these error estimates also improve the performance of the corresponding template structure in MR. A relatively simple modeling protocol seems to be sufficient to provide a model that can be processed with ProQ2 (Ray et al., 2012) and to yield useful error estimates. In fact, it is perhaps important to use a modeling protocol that avoids changing the structure significantly from the starting template, so that the error estimates remain valid for the template as well. Although the outlined procedure cannot be used when multiple template structures are used to create the model, the availability of multiple template structures enables the use of consensus methods for error prediction, which would typically yield more accurate error estimates for each starting template than would be obtained from single-model error prediction methods.

## Experimental Procedures

### Metrics Used for Evaluation

#### GDT_TS

GDT_TS is a global measure of the fractions of Cα atoms that are positioned correctly (Zemla, 2003), and is a score widely used in CASP assessment.

#### LLG

LLG measures how much better than a random model an atomic model explains the measured X-ray amplitudes (Read, 2001). It takes into account the completeness of the model as well as the errors in atomic coordinates. It requires an initial error estimate between the model and the structure, but it is possible to refine this and attain a score independent of this starting value.

#### Vrms

Vrms is the error estimate for a model that gives the optimal (highest) LLG score (Oeffner et al., 2013). It can be thought of as a quantity analogous to an rmsd that is calculated with a distance cutoff, because deviations larger than about half the resolution do not get penalized further (Read, 1990).

### Calculation Steps

#### Translating Errors to Atomic Displacement Parameters

The error estimates are converted to an atomic displacement parameter by squaring and multiplying by 8π^2^/3. This gives a falloff corresponding to the Fourier transform of the assumed gaussian error distribution. However, before the MR calculation the structure factors computed from the model are normalized and therefore the calculation is only affected by the difference between the B factors for regions of high confidence and low confidence, and not by any changes in the overall average B factor.

#### Calculating Log-Likelihood Score

First, the asymmetric unit of the target structure is analyzed. If there are multiple copies of the target protein, a reference chain is selected (typically the most well-ordered) and superposed onto each copy. If large deviations are found among the copies, a selection excluding the variable parts is created manually and the process is repeated (for predictions from the CASP10 experiment, these selections were established by the participants); otherwise all transformations relating the reference chain to the other chains are stored. If there are additional components in the asymmetric unit that are not being predicted, these can be stored for inclusion in the MR calculation. It was found that inclusion of known but unpredicted segments of the structure (e.g., other domains of a multidomain target) increases the sensitivity of the resulting score significantly (Figure 1). This analysis only needs to be done once for each target.

Second, each prediction is superposed onto the reference chain. When the procedure was applied to predictions from the CASP10 experiment, the structures were presuperposed using the program LGA (Zemla, 2003), and no additional superposition was performed. In principle, any superposition procedure that places the prediction within convergence radius of the refinement procedure is sufficient.

Third, the predictions are trimmed down to exclude the variable parts of the target structure, established in the first step.

Fourth, the asymmetric unit is reconstituted from the superposed prediction and the transformations stored in the first step, including the additional components that are not part of the prediction. In this way an approximately constant fraction of the scattering in the asymmetric unit is modeled, irrespective of the number of copies in the asymmetric unit. Rigid-body refinement, including overall B-factor refinement and vrms refinement, is then performed on all chains in the reconstituted model and the LLG score is calculated.

It is assumed that there is a maximum on the LLG surface corresponding to the correct MR solution and that the initial superposition is within radius of convergence for the refinement procedure.

As atomic displacement parameters are an integral part of the calculation, but are currently not being predicted, the LLG score was calculated with three different B-factor values: (1) the original values that were in the B-factor column, (2) converting the values in the B-factor column assuming these are error estimates according to the procedure explained in the previous section, and (3) setting them to a constant value.

#### Comparison with Available Templates

For CASP10 TBM targets, suitable templates were selected from a homology search using HHPred (Söding et al., 2005), which predated the release of the structure by the PDB (Berman et al., 2003). Templates were modified by the program Sculptor (Bunkóczi and Read, 2011) using the sequence alignment from the homology search and were superposed using backbone atoms onto the target chain. Atomic displacement parameters were not modified. These models were then subjected to the procedure applied to calculate the LLG score for predictions.

For CASP10 refinement targets, the starting model made available to predictors was used as the baseline for each target.

#### Cumulative Evaluation

Since LLG scores calculated against different X-ray data sets are not directly comparable, two different comparison schemes were derived.

First, for each target the average and variance of the LLG scores are established, and used in calculating a Z score for each predicted structure (Equation 2). For each predictor, this is then averaged over all targets (Equation 3). This score measures the relative difficulty of each target in light of the received predictions. This score was calculated using all three B-factor schemes as detailed above.

(Equation 2) $$Z_{structure}=\frac{{\mathrm{LLG}}_{structure}-\mu_{LLG}^{target}}{\sigma_{LLG}^{target}}$$

(Equation 3) $$Z_{predictor}=\frac{\sum_{structures\;from\;predictor}Z_{structure}}{n_{structures\;from\;predictor}}$$

The second scheme is based on an improvement score with respect to a baseline score that would be achievable without modeling. First, the LLG score of unpredicted parts is established, and then that of the baseline model with the unpredicted parts. Next, the LLG score is calculated by replacing the baseline model with each prediction in turn. The improvement score (I score) is calculated by subtracting the LLG of the baseline model (including unpredicted structure) from that of the predictions (including unpredicted structure) and dividing by the difference between the LLG of the baseline model and the unpredicted structure alone. For each target, only the best score is taken per predictor and these are then averaged (Equation 4). Separate averages are available for each B-factor scheme and for the best prediction regardless of the B-factor scheme. This scheme tries to measure the performance for MR directly and weights down the results achieved for targets where a relatively good baseline model is available.

(Equation 4) $$I_{predictor}=\frac{\left(\sum_{target}\frac{\left({\mathrm{LLG}}_{best\;from\;predictor}^{target}-{\mathrm{LLG}}_{baseline}^{target}\right)}{\left({\mathrm{LLG}}_{baseline}^{target}-{\mathrm{LLG}}_{unpredicted}^{target}\right)}\right)}{n_{targets\;attempted\;by\;predictor}}$$

## Figures and Tables

**Table 1:** Summary of Results for Groups that Submitted Meaningful Error Estimates, Compared with the Three Best Structure-Only Predictors

Code	Name	% Rms B Factor	I Score Constant B Factor	I Score Rms B Factor	Models above Baseline (%)	Citation
TS026	ProQ2clust	68	−0.304	−0.149	14.5	Ray et al., 2012
TS088	Panther	77	−0.534	−0.426	2.7	Chida et al., 2013
TS130	Pcomb	66	−0.276	−0.098	13.7	Wallner and Elofsson, 2006
TS273	IntFOLD2	81	−0.416	−0.248	6.5	Buenavista et al., 2012
TS277	Bilab-ENABLE	42	−0.429	−0.327	6.0	Ishida et al., 2003
TS280	ProQ2clust2	66	−0.293	−0.122	15.1	Ray et al., 2012
TS285	McGuffin	59	−0.268	−0.153	11.3	Buenavista et al., 2012
TS388	ProQ2	80	−0.308	−0.204	11.2	Ray et al., 2012
TS479	Boniecki_LoCoGRef	55	−0.465	−0.408	7.2	Boniecki et al., 2003
TS498	IntFOLD	48	−0.411	−0.380	6.8	Roche et al., 2011
TS028	YASARA	NA	−0.183		9.8	Krieger et al., 2009
TS301	LEE	NA	−0.200		9.3	Joo et al., 2014
TS330	BAKER-ROSETTASERVER	NA	−0.186		12.4	Leaver-Fay et al., 2011

%Rms B factor is the percentage of models for which B factors calculated from submitted error estimates gave the highest LLG score from all B-factor schemes evaluated. I scores are defined in Equation 4. Models above baseline indicate the percentage of models yielding higher LLG scores than the corresponding baseline structures used in the I score calculation. NA, no data available; rms, root mean square.

**Table 2:** Improvement Scores for Borderline Molecular Replacement Models, Comparing the Effect of Error Estimates Using Structure-Based and Sequence-Based Alignments

Target			Template		Improvement (%)	
Code	No. of Residues	Resolution (Å)	Code	Identity (%)	LSQMAN	MUSCLE
2har	263	1.90	1fby_a	15	80.73	114.79
1w69	390	2.20	2alx_a	19	−8.20	−7.89
1vyg	135	2.40	3elx_a	21	12.49	6.16
1vyg	135	2.40	2f73_a	28	30.89	18.55
1vyg	135	2.40	1crb_a	28	23.00	33.32
1u2y	496	1.95	1bli_a	14	−10.36	58.67
1lke	184	1.90	2hzq_a	21	39.74	73.15
1lke	184	1.90	1z24_a	32	22.63	−1.85
1yhf	115	2.00	2b8m_a	12	28.41	29.55
1ot2	686	2.10	3edd_a	18	−0.24	29.15
1p3c	215	1.50	1mza_a	17	17.81	−21.49
1icn	131	1.74	2ft9_a	30	36.03	23.79
1z07	166	1.81	1r4a_a	20	41.01	72.38
1z07	166	1.81	1zd9_a	23	63.00	74.62
1dzx	215	2.18	2irp_a	23	15.82	24.83
1eem	241	2.00	1fw1_a	22	52.94	31.34
2ikg	316	1.43	1pz1_a	19	66.45	26.14
1t40	316	1.80	1pz1_a	19	80.87	20.99
7taa	478	1.99	3dhu_a	17	25.36	−11.44
1e0s	174	2.28	2eqb_a	16	25.04	−27.30

The resolution column corresponds to the resolution of the data used for the calculation and not the full resolution of the data. Improvement is defined as the difference between the error-weighted LLG, computed using B factors calculated from coordinate errors predicted using ProQ2, and the LLG, computed using constant B factors, normalized by the absolute value of the LLG calculated with the constant B-factor scheme.
